# Effect of the Gas6 c.834+7G>A Polymorphism and the Interaction of Known Risk Factors on AMD Pathogenesis in Hungarian Patients

**DOI:** 10.1371/journal.pone.0050181

**Published:** 2012-11-29

**Authors:** Gergely Losonczy, Attila Vajas, Lili Takács, Erika Dzsudzsák, Ágnes Fekete, Éva Márhoffer, László Kardos, Éva Ajzner, Begoña Hurtado, Pablo Garcia de Frutos, András Berta, István Balogh

**Affiliations:** 1 Department of Ophthalmology, University of Debrecen, Debrecen, Hungary; 2 Department of Laboratory Medicine, University of Debrecen, Debrecen, Hungary; 3 Department of Anesthesiology and Intensive Care, University of Debrecen, Debrecen, Hungary; 4 Hygiene and Infection Control Services, Kenézy Gyula County Hospital, Debrecen, Hungary; 5 Central Laboratory, Jósa András Teaching Hospital, Nyíregyháza, Hungary; 6 Department of Cell Death and Proliferation, Institute of Biomedical Research of Barcelona (IIBB-CSIC, IDIBAPS), Barcelona, Spain; University of Tampere, Finland

## Abstract

Age-related macular degeneration (AMD) is the leading cause of blindness in the elderly in the developed world. Numerous genetic factors contribute to the development of the multifactorial disease. We performed a case-control study to assess the risk conferred by known and candidate genetic polymorphisms on the development of AMD. We searched for genetic interactions and for differences in dry and wet AMD etiology. We enrolled 213 patients with exudative, 67 patients with dry AMD and 106 age and ethnically matched controls. Altogether 12 polymorphisms in Apolipoprotein E, complement factor H, complement factor I, complement component 3, blood coagulation factor XIII, HTRA1, LOC387715, Gas6 and MerTK genes were tested. No association was found between either the exudative or the dry form and the polymorphisms in the Apolipoprotein E, complement factor I, FXIII and MerTK genes. Gas6 c.834+7G>A polymorphism was found to be significantly protective irrespective of other genotypes, reducing the odds of wet type AMD by a half (OR = 0.50, 95%CI: 0.26–0.97, p = 0.04). Multiple regression models revealed an interesting genetic interaction in the dry AMD subgroup. In the absence of C3 risk allele, mutant genotypes of both CFH and HTRA1 behaved as strongly significant risk factors (OR = 7.96, 95%CI: 2.39 = 26.50, p = 0.0007, and OR = 36.02, 95%CI: 3.30–393.02, p = 0.0033, respectively), but reduced to neutrality otherwise. The risk allele of C3 was observed to carry a significant risk in the simultaneous absence of homozygous CFH and HTRA1 polymorphisms only, in which case it was associated with a near-five-fold relative increase in the odds of dry type AMD (OR = 4.93, 95%CI: 1.98–12.25, p = 0.0006). Our results suggest a protective role of Gas6 c.834+7G>A polymorphism in exudative AMD development. In addition, novel genetic interactions were revealed between CFH, HTRA1 and C3 polymorphisms that might contribute to the pathogenesis of dry AMD.

## Introduction

Age-related macular degeneration (AMD) is the leading cause of visual impairment in the elderly in developed countries, most prominently in white populations [1], [2]. The prevalence of the disease increases with age reaching as much as 15% above the age of 80 [3]. AMD is a multifactorial disease characterized by progressive degeneration of the central retina leading to visual deterioration. Early stages of AMD are characterized by retinal pigment abnormalities and accumulation of small deposits called drusen under the macular area of the retina. Two advanced forms of the disease are distinguished: extensive pigment epithelium atrophy also referred to as geographic atrophy (GA) leading to irreversible and untreatable visual deterioration or subretinal neovascular membrane (SRNVM) formation characterized by intra- or subretinal invasion of new vessels arising from the choroid. Multiple methods have been developed to determine the severity of the disease and to estimate the risk of progression from early to late stages. The neovascular form is referred to as exudative or wet type, while AMD without any neovascular component is considered non-exudative or dry type AMD [4]. Although the etiology of the disease is still largely unclear, several risk factors have been unequivocally linked to AMD, including advanced age, European origin and smoking. Other factors including high cholesterol level, sunlight exposure, blue iris, oxidative damage, hypertension, obesity and inflammation have also been linked to AMD development, however, with inconsistent results [5], [6]. Cataract surgery has been shown to increase risk exclusively of dry late AMD, however carriers of two complement factor H (CFH) risk alleles are prone to develop all types of AMD [7].

In recent years a growing number of evidence has supported the role of genetic risk factors in AMD pathogenesis. The most widely studied and strongest genetic risk factors map to genes of the alternative complement pathway and its regulators, and are most likely responsible for an imbalance in the complement activation. Additionally, there is a wide range of chromosome regions and genes with different functions implicated in AMD pathogenesis. Because in most studies sharp clinical difference between cases and controls is ascertained by recruiting cases with unequivocally advanced phenotype, little is known about the genetic background of intermediate dry AMD. It is still an open question what makes the difference in the genetic etiology of exudative and non-exudative AMD [8].

After the initial reports [9], [10], [11], numerous case-control studies have provided supportive evidence for a strong association between AMD and the p.Tyr402His (rs1061170) polymorphism of the CFH gene in different populations. According to a very recent meta-analysis of 24 case-control studies the p.Tyr402His variant confers a 2-fold higher risk of late-AMD per copy in individuals of European descent [12].

Other components of the complement system have also been implicated in AMD pathogenesis. The initial report demonstrated that the p.Arg102Gly (rs2230199) polymorphism in the Complement factor 3 (C3) gene confers statistically significant risk to AMD with an odds ratio of 1.7 for heterozygotes and 2.6 for homozygotes in comparison to wild type subjects [13]. Later similar odds ratios were reported equally contributing to early and late AMD. The risk was independent of CFH p.Tyr402His and LOC387715 p.Ala69Ser polymorphisms [14], [15].

The rs10033900 polymorphism of Complement factor I (CFI), a proteolytic regulatory enzyme inactivating C3b has been shown to be associated with AMD [16], [17], however a recent paper failed to confirm this association [18].

Strong association has been confirmed between AMD and the p.Ala69Ser (rs10490924) polymorphism in LOC387715 coding a protein of unknown function [19], [20] or a promoter SNP of the HTRA serine peptidase 1 (HTRA1) gene (rs11200638) [21], [22], [23], [24]. These two polymorphisms are located in close vicinity and it is still a subject of debate which of the two plays a role in the pathomechanism of the disease.

Although lipids are major components of drusen, proteins involved in lipid transportation have been linked to AMD pathogenesis only recently [25]. Apolipoprotein E (ApoE), a polymorphic gene with three common allelic variants (E2, E3 and E4) has also been connected to the pathogenesis of AMD. E3 is the major allele among whites. E4 allele has been reported to decrease AMD risk, or at least delay the occurrence of the disease. In contrast, the presence of the E2 allele is associated with increased risk and younger age at diagnosis [26], [27], [28]. ApoE E4 proved to be protective with an odds ratio of 0.5 (95%CI: 0.29–0.86) in a study comprising of 3137 individuals [29]. Another study demonstrated a protective effect of the E4 allele only in the neovascular AMD patient group with an OR of 0.61 (95%CI: 0.38–0.97) [30]. In contrast with these findings, a Spanish study reported an increased risk for AMD due to the ApoE E4 allele, with an OR of 5.6 [31]. Previously we could not demonstrate statistically significant association between ApoE alleles and AMD in a Hungarian population. However, the potential risk factor E2 allele was less frequent in patients than in controls (0.066 and 0.1, respectively) while the E4 allele was more frequent in the patient group than in controls (0.108 versus 0.084, respectively), interestingly [32]. The somewhat inconsistent findings on the association of AMD and ApoE called for a recently published pooled analysis (n = 21.160) demonstrating that the E2 allele in homozygous form confers risk (OR = 1.83, 95%CI: 1.04–3.23) and the E4 allele is protective (OR = 0.72 per haplotype; 95%CI: 0.65–0.74) in late AMD [33]. A recent well powered case-control study (including 2187 cases and 2187 age and ethnically matched controls) demonstrated disease risk of the E2 allele compared to the E3/E3 genotype (OR = 1.32, 95%CI: 1.11–1.58). However, the E2 allele only conferred risk to early AMD in the never smoker and previously smoker group, while the E4 allele was protective against early AMD exclusively in the current smoker population [34].

The growth arrest–specific gene 6 (Gas6) product is a vitamin K dependent protein secreted by leukocytes and endothelial cells in response to injury. Gas6 has a high structural homology with the natural anticoagulant protein S. It has not been implicated in AMD pathogenesis so far, however there are certain functions of the protein that make it a possible candidate for AMD. It has growth factor-like properties through its interaction with receptor tyrosine kinases of the TAM family; Tyro3, Axl and MerTK, this way it contributes to the regulation of angiogenesis, cell survival, proliferation, migration and adhesion, making it a relevant participant of biological processes like atherogenesis and thrombosis [35], [36], [37], [38]. Recently, we found that Gas6 is present in the human circulation [39]. A Gas6 polymorphism (c.834+7AA genotype) was identified as a common SNP in the general population and gave the strongest association with stroke [40]. Others found similar association with acute coronary syndrome, and type 2 diabetes [36], [40], [41]. TAM receptor tyrosine kinases and their ligands Gas6 and Protein S are essential for the phagocytosis of apoptotic cells and membranes in the immune, nervous, and reproductive systems [42]. Moreover, MerTK receptor is the key player involved in photoreceptor outer segment phagocytosis by RPE cells. MerTK knock-out mice develop almost total degeneration of the retinal photoreceptor layer by 10 weeks of age [43], [44], [45]. MerTK SNPs were selected because of their tagging properties after Haploview analysis of the genotypes downloaded from the HapMap database for CEU population (Rel20/phase II Jan06, on NCBI B35 assembly, dbSNP b125). Haplotype (defined as a group of closely linked polymorphic alleles in a chromosomal region) tagging SNPs were selected according to the following parameters: MAF>0.05 in Caucasians, r2>0.8 between each pair of tagged and tagging SNPs in pairwise tagging and haplotypes coverage down to 5% of their frequency in the HapMap CEU population for each analysed gene. The 4 tagSNPs selected belong to a haplotype block and were sufficient to cover the whole gene [46].

The aim of our present case-control study was to refine the association of the known polymorphisms and AMD subtypes in the Hungarian population and to explore the interplay of different polymorphisms. We considered MerTK and its ligand Gas6 as candidate genes to play a role in AMD pathogenesis and searched for association between their polymorphisms and the disease.

## Patients and Methods

### Ethics Statement

The study was approved by the Institutional Ethics Committee of the University of Debrecen and the procedures strictly adhered to the tenets of the declaration of Helsinki.

### Patients

In total, 386 subjects were enrolled in the case-control study: 213 patients with exudative AMD, 67 patients with dry AMD and 106 unrelated ethnically matched healthy controls were ascertained in Eastern-Hungary at the Department of Ophthalmology, University of Debrecen between 2005 and 2011. The control group consisted of individuals attending to the Outpatient Clinic because of refractive disorders, or post-cataract follow-up visits. To maximize the reliability of our study, we deliberately selected controls to be somewhat older than patients. Written informed consent was obtained from all participants. Detailed patient history was recorded with the use of a questionnaire focusing on known or suspected non-genetic risk factors of AMD such as cigarette smoking, exposure to blue light, medical history of acute myocardial infarction, ischemic heart disease and deep venous thrombosis. Cigarette smoking was determined in packyears. Smoking of one pack of cigarettes daily for a period of one year was considered one packyear. Body mass index (BMI) was calculated on the actual body height and weight values when ascertained that no considerable change in body weight occurred in the past 10 years. An average BMI was calculated in patients with significant loss or increase of body weight based on body weight values of the previous 10 years period. Color fundus photographs were taken of patients and controls. Fluorescein angiography was used to investigate patients with exudative AMD. Grading of the severity of the disease was based on the clinical age-related maculopathy staging system (CARMS) [4]. There were 5,3,18,6 and 35 patients in the dry AMD group characterized as CARMS grade 2b, 2c, 3a, 3b and 4, respectively.

For statistical analysis, patients were classified according to the more severely affected eye into two groups corresponding to either dry or wet AMD. No signs of AMD, such as abnormal pigmentation or drusen were observed in controls. Color fundus photographs and fluorescein angiograms were evaluated by two experienced ophthalmologist (T.L. and L.G.). Patients or controls with other ocular diseases interfering with reliable evaluation of AMD were not included in the study.

### Molecular genetic methods

Molecular genetic methods for the detection of the common ApoE alleles, CFH p.Tyr402His polymorphism, LOC387715 rs10490924 (p.A69S) and HTRA1 rs11200638 polymorphisms were used as described earlier [32]. Factor XIII Val34Leu polymorphism was detected using fluorescent PCR and hybridization probes [47]. Similar methodology was used for the genotyping of Gas6 rs8191974 (c.834+7G>A) polymorphism and for the MerTK rs86016 (g.2920G>A) polymorphism, as described earlier [46]. Complement C3 rs2230199 (p.Arg102Gly) polymorphism was tested using a PCR-RFLP method. Amplification was performed using the primers: C3R102F: 5′- CAG GGA GTT CAA GTC AGA AAA GG -3′ and C3R102R: TCT TGT CTG TCT GGA TGA AGA GG -3′. The 131 bp PCR product was then subjected to the digestion with *Cfo*I restriction endonuclease. The sole restriction site of the enzyme in the PCR product is lost when the mutation is present. The candidate, most likely a tag SNP, that has been shown to be associated with AMD, rs10033900 in the complement factor I (CFI) gene was tested using TaqMan SNP Genotyping assay. TaqMan assays were also used for the detection of the MerTK I1b rs17835605 (g.4916C>T), MerTK I1c rs10496440 (g.8809A>C) and MerTK I4 rs7573344 (g.60127A>G), as described earlier [46]. Over 98% of controls and 96% of patients were genotyped in each test. For the verification of all in-house mutation detection methods described above, randomly selected samples were sequenced with the same primers that were used for PCR amplification. No discrepancy was found in any of the tested samples.

### Statistical methods

Biallelic polymorphisms were tested using the chi-square test for the deviation of Hardy-Weinberg equilibrium in the control group. Continuous and categorical variables were described in terms of mean (SD) and frequency (%), respectively, in AMD subtype groups and in controls. Unadjusted between-group comparisons were made using ANOVA or Kruskal–Wallis tests subject to normality and homoscedasticity assumptions (continuous variables), and Fisher's exact test (categorical variables). Unadjusted effects of explanatory variables were estimated using simple logistic regression and expressed as odds ratios and 95% confidence intervals (CI). Continuous variables were transformed with the formula providing the best achievable fit. Categorical variables were regrouped by pooling groups with close to identical effects if applicable. Models were fitted for the total sample and for subsamples formed by restricting cases to dry or wet AMD variants. Adjusted effects were estimated using multiple logistic regression. The significance criterion was set at α = 0.05 for all tests, i.e. with no formal adjustment for multiple testing. Categorical variables were regrouped by pooling groups with close to identical effects if applicable. Age and cataract surgery were included as a priori adjustment covariates without effect interpretations, due to their association with study group inherent in the control recruitment methods used. Further variable selection was based on an iterative procedure where models were started by including all explanatory variables with significant unadjusted effects, adding each of the rest one by one and keeping if meaningful, and were made parsimonious by eliminating those of neutral behavior. Possible interactions between explanatory variables of models thus developed were systematically evaluated and kept if found significant. For comparability between dry and wet AMD variant models, genetic interactions included in any subtype model were also used in the other. Model fit was checked using Hosmer–Lemeshow tests for all three final models. As HTRA1 rs11200638 and LOC rs10490924 are in linkage disequilibrium, for the analysis HTRA1 rs11200638 was selected.

## Results

### Characteristics of the study population

In total, 280 patients and 106 controls were enrolled in the present study. Basic characteristics of the study population are summarized in Table 1. Patients were divided into two subgroups according to disease phenotype involving 213 patients in the wet and 67 patients in the dry AMD subgroups. There was a slight female predominance among wet AMD patients which is in line with European data on gender distribution in neovascular AMD [48].

**Table 1:** Characteristics of the study population.

Attributions	Dry AMD	Wet AMD	Controls	p
	N = 67	N = 213	N = 106	
**Female N (%)**	34 (50.7)	125 (58.7)	53 (50.0)	0.26
**Male N (%)**	33 (49.3)	88 (41.3)	53 (50.0)	0.26
**Age (year, mean±SD)**	75.4 (11.5)	76.0 (7.3)	79.1 (6.1)	0.005
**Smoking (packyear, mean± SD)**	12.0 (20.1)	11.2 (19.8)	11.4 (21.9)	0.76
**BMI (mean±SD)**	27.3 (4.5)	27.3 (4.4)	27.6 (4.7)	0.83
**Hypertension N (%)**	45 (67.2)	159 (75.0)	81 (79.4)	0.21
**DVT N (%)**	7 (10.4)	15 (7.1)	15 (14.9)	0.09
**AMI N (%)**	6 (9.0)	19 (9.0)	7 (7.4)	0.90
**Outdoor profession N (%)**	22 (32.8)	39 (18.5)	38 (38.4)	0.0004
**Cataract surgery N (%)**	21 (31.3)	48 (22.6)	73 (70.2)	<0.0001

BMI: body mass index, DVT: deep vein thrombosis, AMI: acute myocardial infarction.

There was no significant difference between the ages of the dry and wet subgroups of patients, however, controls were significantly older than patients as a result of intentionally older control selection. Cataract surgery was also more common in the control group because of controls selection methods and probably because the older age of the controls. There was no statistically significant difference between patients and controls in terms of frequency of hypertension, deep venous thrombosis, myocardial infarct, smoking and body mass index. Interestingly, outdoor profession was significantly more frequent among controls than patients.

### Genotype distribution and unadjusted odds ratios of the analyzed polymorphisms

The genotype distributions in controls and patients are shown in Table S1. Actual sample number is provided for each test. Small differences are due to the shortage of DNA sample quantity available. The control group showed no statistical difference from Hardy-Weinberg equilibrium in the case of the tested polymorphisms. Minor alleles of genes unequivocally linked to AMD were more frequent in patients than in controls. Except I1a, MerTK receptor polymorphisms showed a very low minor allele frequency and no notable difference between cases and controls. In case of ApoE the most common E3 allele was slightly more frequent in patients than in controls while the potential risk factor E2 allele was less frequent in patients than in controls. The ApoE E4 allele had similar frequency in all the three groups. Unadjusted odds ratios, and corresponding p values for each genotype comparison are shown in Table 2.

**Table 2:** Unadjusted ORs of the examined polymorphisms.

Polymorphism	Genotypes compared	Dry AMD		Wet AMD	
		OR	p value	OR	p value
**CFH rs1061170**	TC+CC vs. TT	1.30	0.43	3.27	<0.0001
**CFH rs1061170**	TC vs. TT	0.86	0.67	2.09	0.0104
**CFH rs1061170**	CC vs. TC	3.86	0.003	4.17	0.0001
**CFH rs1061170**	CC vs. TT	3.32	0.01	8.73	<0.0001
**CFH rs1061170**	CC vs. TT+TC	3.60	0.002	5.45	<0.0001
**LOC387715 rs10490924**	GT+TT vs. GG	1.50	0.20	3.29	<0.0001
**LOC387715 rs10490924**	GT vs. GG	1.22	0.56	2.07	0.01
**LOC387715 rs10490924**	TT vs. GT	3.55	0.03	7.47	<0.0001
**LOC387715 rs10490924**	TT vs. GG	4.31	0.01	15.5	<0.0001
**LOC387715 rs10490924**	TT vs. GG+GT	3.89	0.01	10.0	<0.0001
**HTRA1 rs11200638**	TC+CC vs.TT	1.52	0.19	3.21	<0.0001
**HTRA1 rs11200638**	TC vs. CC	1.24	0.52	2.00	0.01
**HTRA1 rs11200638**	CC vs. TC	4.03	0.03	9.18	<0.0001
**HTRA1 rs11200638**	CC vs. TT	5.00	0.01	18.4	<0.0001
**HTRA1 rs11200638**	CC vs. TT+TC	4.46	0.01	12.2	<0.0001
**Gas6 rs8191974**	AA vs. GA	1.51	0.39	1.00	0.99
**Gas6 rs8191974**	AA vs. GG	1.03	0.96	0.61	0.21
**Gas6 rs8191974**	AA vs. GG+GA	1.27	0.60	0.79	0.54
**Gas6 rs8191974**	GA vs. GG	0.68	0.26	0.61	0.05
**Gas6 rs8191974**	GA+AA vs. GG	0.75	0.37	0.61	0.04
**C3 rs2230199**	CG+GG vs. CC	1.55	0.17	1.27	0.3237
**C3 rs2230199**	CG vs. CC	1.47	0.24	1.22	0.4198
**C3 rs2230199**	GG vs. CG	1.76	0.48	1.52	0.5409
**C3 rs2230199**	GG vs. CC	2.59	0.23	1.86	0.3568
**C3 rs2230199**	GG vs. CC+GC	2.22	0.31	1.73	0.41
**CFI rs10033900**	CC vs. TC	1.47	0.34	1.21	0.55
**CFI rs10033900**	CC vs. TT	1.54	0.34	1.31	0.44
**CFI rs10033900**	TC vs. TT	1.04	0.91	1.09	0.76
**FXIII rs5985**	GT+TT vs. GG	0.59	0.10	0.66	0.08
**FXIII rs5985**	GT vs. GG	0.65	0.20	0.64	0.08
**FXIII rs5985**	TT vs. GT	0.51	0.34	1.15	0.73
**FXIII rs5985**	TT vs. GG	0.34	0.11	0.74	0.46
**FXIII rs5985**	TT vs. GG+GT	0.40	0.18	0.90	0.78
**Apo-E**	3/4+4 vs. 2+2/3	1.67	0.3897	1.51	0.3512
**Apo-E**	3/4+4 vs. 3/3	1.00	0.9929	1.02	0.9543
**Apo-E**	2+2/3 vs. 3/3	0.60	0.2879	0.68	0.2632
**Mertk I1a**	AA vs. GA	0.96	0.9251	1.08	0.8171
**Mertk I1a**	AA vs. GG	0.83	0.7086	1.00	0.9910
**Mertk I1a**	GA vs. GG	0.87	0.6891	0.93	0.7714
**Mertk I1b**	CT vs. CC	0.58	0.1329	0.76	0.2919
**Mertk I1b**	TT vs. CC	2.50	0.0989	1.16	0.7664
**Mertk I1b**	TT vs. CT	4.33	0.0144	1.52	0.4218
**Mertk I1c**	AC vs. AA	0.54	0.2623	1.13	0.7328
**Mertk I4**	AG vs. AA	0.88	0.7186	0.79	0.3602
**Mertk I4**	GG vs. AA	*	*	0.62	0.5352
**Mertk I4**	GG vs. AG	*	*	0.79	0.7615

* Missing data are due to insufficient minor allele frequency.

### Multiple regression models

The model of pooled dry and wet AMD variants revealed strongly significant adjusted risks in case of the presence of homozygous polymorphisms in the CFH and HTRA1 genes, increasing the odds of AMD by more than five and nearly ten times (OR = 5.3, 95%CI: 2.5–11.5, p<0.0001, and OR = 9.8, 95%CI: 3.3–29.5, p<0.0001), respectively. No significant interactions were found between any pairs of these variables.

**Figure 1:**
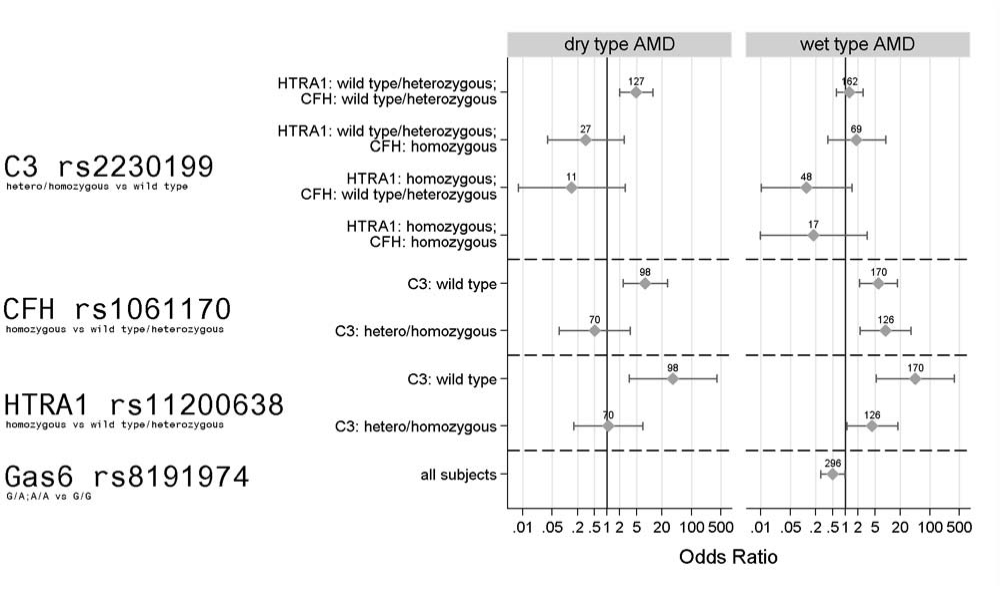
The effect of GAS6 c.834+7G>A polymorphism and genetic interactions on the risk of AMD. Polymorphisms with the compared genotypes are shown on the left side. Stratum of subjects involved in the corresponding analysis are on the vertical axis. Figures above markers indicate number of observations for the given stratum Adjusted odds ratios and 95% confidence intervals are represented by dots and lines, respectively. In the dry AMD group the effect of C3 polymorphism in case of double CFH/HTRA1 homozygousity could not be estimated due to low observation number. GAS6 was not included as an explanatory variable in the model for dry AMD.

Factors showing significant adjusted associations with dry type AMD are shown in Table 3. and Figure 1. These are genes C3, CFH, and HTRA1, with interactions between both C3 and CFH (p = 0.020) and C3 and HTRA1 (p = 0.024). The heterozygous/homozygous genotype of C3 carried a significant risk over wild type genotype in the simultaneous absence of homozygous CFH and HTRA1 polymorphisms only. In this case it was associated with a near-five-fold relative increase in the odds of dry type AMD. The stratum of subjects with homozygous mutated HTRA1 and CFH genes contained an insufficient number and distribution of observations for C3 effect estimation. In the case of wild type C3 genotype, mutated alleles of both CFH and HTRA1 behaved as strongly significant risk factors, but reduced to neutrality otherwise.

**Table 3:** Polymorphisms showing significant association with dry AMD using multiple logistic regression.

Polymorphism	Genotypes compared	Stratum of subjects	OR	95% CI	p value
**C3 rs2230199**	CG+GG vs. CC	**HTRA1**: TT+TC **CFH**: TT+TC	4.93	1.98–12.25	0.0006
**CFH rs1061170**	CC vs. TT+TC	**C3**: CC	7.96	2.39–26.50	0.0007
**HTRA1 rs11200638**	TC+CC vs.TT	**C3**: CC	36.02	3.30–393.02	0.0033

Stratum represents genotype of subjects involved in the analysis.

Factors found to have remarkable associations with wet type AMD included the genes Gas6, CFH, and HTRA1 (Table 4, Figure 1). Gas6 was protective irrespective of other genotypes (p = 0.04), reducing the odds of wet type AMD by a half. There was no evidence for the effect of C3 (left in the model for comparability with the dry variant), and although C3's odds ratios specific for different combinations of CFH and HTRA1 genotypes showed a fairly varied pattern, the interaction was technically not significant, unlike in the case of the dry AMD. However, there was some indication that the effect of HTRA1 (homozygous vs. wild type or heterozygous genotype) was modified by the C3 genotype, with estimated odds ratios of 45.04 (p = 0.0005) and 4.34 (p = 0.04) in C3 wild type and hetero/homozygous mutated genotypes, respectively. Homozygous CFH polymorphism was observed to be a highly significant risk factor for wet type AMD, with odds ratios slightly varying around 7.5 depending on whether mutations in C3 were present or not. All models had a sufficient goodness-of-fit by Hosmer–Lemeshow tests.

**Table 4:** Polymorphisms showing significant association with wet AMD using multiple logistic regression.

Polymorphism	Genotypes compared	Stratum of subjects	OR	95% CI	p value
**CFH rs1061170**	CC vs. TT+TC	**C3**: CC	6.08	2.17–17.04	0.0006
**CFH rs1061170**	CC vs. TT+TC	**C3**: CT+TT	8.96	2.24–35.79	0.002
**HTRA1 rs11200638**	TC+CC vs.TT	**C3**: CC	45.04	5.30–382.83	0.0005
**HTRA1 rs11200638**	TC vs. CC	**C3**: CT+TT	4.34	1.09–17.28	0.04
**Gas6 rs8191974**	GA+AA vs. GG	n.a.	0.5	0.26–0.97	0.04

Stratum represents genotype of subjects involved in the analysis.

## Discussion

Our understanding about the genetic causes and therefore pathological pathways involved in the development of AMD has increased substantially in the last few years. The p.Tyr402His polymorphisms of the CFH gene, the rs11200638 polymorphism of the serine peptidase HTRA1 and the p.Ala69Ser polymorphism of the LOC387715 gene are unequivocally associated to AMD in different populations with robust risks. Our results further support these observations and demonstrate robust and highly significant adjusted risk conferred by these polymorphisms as shown by the analysis of the pooled AMD population. After dividing the patient group to exudative (wet) and non-exudative (dry) subgroups, other associations and an interesting genetic interplay was revealed. The rs2230199 polymorphism of the C3 gene contributed significant disease risk to non-exudative AMD in the simultaneous absence of homozygous CFH and HTRA1 polymorphisms. Alternative complement activation is the major biological process linked to AMD development so far. C3 plays a central role in the alternative pathway and its role in AMD pathogenesis is supported by epidemiological and experimental observations. The C3 polymorphism had no significant effect on the development of wet AMD in our study population.

Epidemiological reports on the p.Arg102Gly C3 polymorphism are usually limited to advanced cases including neovascular AMD and geographic atrophy. This approach, however, falls short of detecting any difference between the etiology of dry and wet AMD. Despite the fact that CFH and C3 are members of the same biological pathway, no evidence of a genetic interplay between these two has been demonstrated so far [49]. Carriers of two risk alleles of the rs2230199 polymorphism of the C3 gene are at moderate risk to develop AMD compared to wild type subjects (OR: 1.88, 95%CI: 1.59–2.23) according to a recent meta-analysis [15]. Besides that, it is well known that, drusen, the hallmark of dry AMD contains complement factors 3a and 5a among other inflammatory proteins, which therefore could play a role in drusen formation and might even have an effect on neovascularisation [50], [51], [52], [53], [54]. Experimental evidence showed that suppression of the complement cascade in the retina delayed and reversed the onset of AMD in a monkey model of dry AMD [55]. The effect of CFH and C3 polymorphisms are mutually exclusive on the risk of dry AMD in our study population. On the other hand, CFH has a robust effect on neovascular AMD independent of the C3 status. Since the two proteins are located in the same biological pathway, our results can be explained with the stronger effect overriding the other. Interestingly, the interaction between C3 and HTRA1 is very similar, which indicates that C3 and HTRA1 are also competing in dry AMD development. This finding underscores previous reports on the role of HTRA1 in complement regulation [56]. Taken together our findings indicate a central role of C3 in dry AMD formation, however, CFH and HTRA1 genotype have an influence on the effect of the C3 p.Arg102Gly polymorphism most likely explained by their complement regulatory activity. CFI has been associated to AMD development with conflicting results. Fagerness et al. reported an OR of 0.7 for the lower risk C allele (p = 6.46×10^−8^) of the rs10033900 polymorphism within the CFI gene in a large population of European descent [16]. Chen et al found a statistically significant association of the rs2285714 risk allele and AMD with a moderate OR of 1.31 (95%CI: 1.18–1.45, p = 3.4×10^−7^) [57]. Seddon et al. demonstrated that the protective effect of the hepatic lipase (LIPC) rs10468017 polymorphism on AMD pathogenesis was stronger in the presence of double wild type rs10033900 compared to the homozygous polymorphism [58]. The findings of the original article by Fagerness were reflected in three case-controls studies. A report on a Japanese population demonstrated an OR of 0.28 (95%CI: 0.11–0.69, p = 0.0035) for the rare homozygous CC genotype [59]. The work of Ennis et al. supports the involvement of the CFI gene in AMD development by demonstrating four SNP's in the CFI gene associating to AMD, however the effect of the rs10033900 polymorphism, which had the strongest association signal in the original work of Fagerness, was not statistically significant in their cohort (p = 0.135). Interestingly, the haplotype including rs10033900 polymorphism and another non-significant polymorphism together conferred statistically significant disease risk (OR = 2.15, p = 0.02) [17]. A very well powered case-control study based on independent samples from England and Scotland failed to detect any statistically significant effect of the rs10033900 polymorphism on AMD (OR = 0.95, 95%CI: 0.83–1.09, p = 0.47) [18]. Similarly to that, in the present study we could not provide any additional support for the association of the rs10033900 polymorphism and AMD. Although our sample size is not as large as some of the cited works, it is large enough to detect strong or even weak but significant associations with other well established risk factors of AMD as demonstrated by our findings on the CFH, LOC387715, HTRA1 and C3 polymorphisms. Our results are in line with studies showing no effect of the rs10033900 polymorphism. Although one cannot exclude such an association based on a single negative result, there are a growing number of publications falling short of detecting any effect of the polymorphism which clearly indicates that the association is not unequivocal and calls for further epidemiological and functional analysis to clarify the link between CFI and AMD.

No evidence of association with AMD could be demonstrated in the case of ApoE alleles. We neither could confirm previous reports on the protective effect of the E4 allele, nor could we show any risk related to the E2 allele. Two recent very-well powered epidemiological studies [33], [34] concluded on the disease causing effect of the E2 allele and the protective role of the E4 allele. Albeit these convincing studies, our results still do not fit in the line. Interestingly, we could not observe any statistically significant disease association with any of the ApoE alleles, moreover the frequency of the E2 risk allele was higher in controls than in patients, while the protective E4 allele showed similar frequencies in the wet AMD and the control group. These data indicate that ApoE is not a key player in AMD pathogenesis in the Hungarian population. This can either be explained by a geographical difference, or by other different environmental factors, probably the different smoking habits of the Hungarian population.

Candidate gene approach is widely used to identify biological pathways and genes involved in pathological processes. Based on their biological functions, we directly tested for disease association of common polymorphisms in the FXIII, Gas6 and in one of the Gas6 receptor genes, MerTK. Blood coagulation factor XIII is a plasma transglutaminase that cross links adjacent fibrin chains in the final step of the coagulation cascade. In addition, FXIII is known to participate in wound healing, tissue remodeling and embryo implantation at least partially through its proangiogenic effect [60]. The cardioprotective p.Val34Leu polymorphism of the blood coagulation factor XIII has been associated with accelerated thrombin activation, recurrent subconjunctival haemorrhage, intracerebral haemorrhage, and a decreased immune reaction in humans [61], [62], [63]. Most importantly, since angiogenesis plays a crucial role in neovascular AMD, we found it reasonable to investigate if the polymorphism had any effect on AMD formation. However, we failed to detect any association of AMD and the polymorphism in our present study, indicating a neutral polymorphism in relation to both dry and wet AMD development.

Gas6, a Protein S structural homologue, has important functions in the regulation of angiogenesis, cell migration and proliferation. Its common polymorphism Gas6 c.834+7G>A is associated with decreased risk of cardiovascular diseases. Its receptors (TAM) play a crucial role in phagocytosis of apoptotic cells in the immune, nervous, and reproductive systems. These functions make Gas6 and its receptors a potential candidate involved in both dry and wet AMD development. In the pooled early and late AMD population the Gas6 polymorphism showed no statistically significant association to AMD. When analyzing dry and wet subgroups separately, no association with the dry AMD could be demonstrated; however a protective effect was detected in the wet AMD subgroup (p = 0.04). As formation of new vessels is a hallmark of wet AMD, it is likely that it is this function of Gas6 that is important in the context of the disease. Gas6 is able to inhibit VEGF-A signaling through Axl activation of SHP-2 phosphatases [38]. Recently, a similar inhibitory effect has been shown in metastasis induced angiogenesis [64]. This finding suggests a role of Gas6 in controlling pathological angiogenesis, and that c.834+7G>A polymorphism of the GAS6 gene, which has been linked to disease repeatedly [36], [40], [41], could have a direct effect on the function of the gene through an unknown mechanism. Future epidemiological and functional studies are required to further evaluate the effect of the GAS6 polymorphism on AMD development. It is to be noted, that no formal correction for multiple testing was applied in the multiple logistic regression analysis, however, this does not invalidate the conclusion that the suggested effect of the GAS6 polymorphism is worthy of further investigations.

In our case-control study we confirmed previous findings on the association of AMD and genetic polymorphisms in the CFH, LOC387715 and HTRA1 genes, however could not demonstrate any association with the CFI gene. More interestingly, we found a genetic interplay of CFH, HTRA1 and C3 genes, showing that the C3 polymorphism is a major contributor of dry AMD in the absence of the other polymorphisms, while has no effect on wet AMD development in our population. This result indicates that C3 plays a critical role in dry AMD rather than wet AMD pathogenesis, and its effect can be overdriven by other known genetic risk factors in the CFH and HTRA1 genes. We detected a protective effect of a common Gas6 c.834+7G>A polymorphism on wet AMD formation. Our results suggest a possible new player and novel genetic interactions in AMD pathogenesis, however our data should be confirmed in other populations, especially in the case of the Gas6 polymorphism.

## Supporting Information

Table S1 Genotype distribution of the analyzed polymorphisms in patients and controls.
(XLSX)
